# Role of dihydrotestosterone in whole-body energy utilization during acute running exercise in mice

**DOI:** 10.20463/jenb.2018.0010

**Published:** 2018-06-30

**Authors:** Nahyun Kim, Jisu Kim, Kiwon Lim, Jonghoon Park

**Affiliations:** 1 Department of Physical Education, Korea University, Seoul Republic of Korea; 2 Laboratory of Exercise Nutrition, Department of Physical Education, Konkuk University, Seoul Republic of Korea

**Keywords:** Exercise, dihydrotestosterone, energy expenditure, carbohydrate oxidation, fat oxidation

## Abstract

**[Purpose]:**

Dihydrotestosterone (DHT) plays an important role in various processes that utilize energy, including fat and carbohydrate oxidation. The purpose of this study was to determine the effect of inhibiting DHT formation during mid-intensity running exercise on energy expenditure and fat and carbohydrate oxidation in the whole body using a calorimetric chamber.

**[Methods]:**

Twelve ICR adult male mice, 9 weeks of age, were randomized into two groups: CON (n = 6, no treatment with exercise) and CONIN (n = 6, DHT inhibitor treatment with exercise, SRD5A1A2 is an enzyme involved in the metabolism of free testosterone into DHT). Inhibitor was administered to the CONIN group intraperitoneally, while the CON group was treated with vehicle (corn oil 2 mg/kg). After 3 days of administration of the inhibitor or vehicle, exercise was performed at 60–70% VO_2_max for 30 min on a treadmill in a calorimetric chamber. The O_2_ uptake, CO_2_ production, carbohydrate and fat oxidation, and respiratory exchange ratio (RER) during 30-min exercise were measured using a calorimeter.

**[Results]:**

During a single bout of exercise, the CONIN group showed a significantly higher area under the curve (AUC) of O_2_ uptake and CO_2_ production from 20 min into exercise than the CON group (*p* < 0.001). The CONIN group showed a significantly higher AUC for carbohydrate oxidation from 20 min into exercise than the CON group (*p* < 0.001), whereas no difference was found in fat oxidation between groups (*p* = 0.067). The CONIN group had a significantly higher AUC of RER from 20 min into exercise than the CON group (*p* < 0.001).

**[Conclusion]:**

We observed increased energy consumption at the later phase of 30-min moderate-intensity treadmill running when DHT production was inhibited. Furthermore, when DHT production during exercise was inhibited, whole-body fat utilization was inhibited and carbohydrate oxidation was substantially increased at the later phase of exercise compared to in the control group. Therefore, changes in DHT concentration in the body during exercise may be involved in whole-body fat utilization, suggesting that DHT may be an important factor affecting endurance exercise capacity.

## INTRODUCTION

Dehydroepiandrosterone (DHEA), a well-known precursor of sex hormones, is metabolized to testosterone through the activity of 17β-hydroxysteroid dehydrogenase, a sex-hormone-synthesizing hormone. Testosterone is converted to dihydrotestosterone (DHT) as a result of elevated 5α-reductase expression (typically SRD5A1 and SRD5A2)^1,2^. DNA information is delivered to the nucleus as testosterone and DHT binds to androgen receptors, based on which essential proteins are produced^1,2^. DHT has a higher affinity for androgen receptors than does testosterone, and DHT is known as the most active androgen hormone^3^. Androgen receptors are present in muscles and brown adipose tissues involved in energy consumption and energy substrate utilization^4,5^, and androgen receptor knock-out mice show reduced energy consumption and lipolysis inhibition^6,7^. Therefore, androgen hormones are thought to be involved in energy metabolism in the body, but the effects of altered androgen hormones energy metabolism during exercise remain largely unknown.

Aizawa et al.^5^ subjected 10-week-old male rats to one-time moderate-intensity treadmill running and analyzed the activities of sex hormones and sex hormone-synthesizing enzymes in the gastrocnemius muscle. They found elevations in the testosterone and DHT concentrations in the muscle and a particularly significant increase in the levels of 5α-reductase type 1 protein, a protein required to convert testosterone to DHT. Moreover, Aizawa et al.^8^ reported that prolonged moderate-intensity running leads to elevations in sex hormone synthesis in the skeletal muscles, particularly 5α-reductase type 1 expression and DHT concentration. In a human study, one-time moderate-intensity ergometer exercise at 70% VO_2max_ significantly increased the concentration of DHEA, a precursor of DHT, in the blood^9^, suggesting that moderate-intensity aerobic exercise results in the production and secretion of androgen hormones in the body.

An increase in DHT concentration was reported to increase beta-oxidation activity in fat tissues and lipolysis^10^. Furthermore, long-term DHT administration has been reported to up-regulate the expression of the gene that transports fat into the mitochondria^11^. Therefore, elevated DHT in the body may improve fat oxidation. Although it is well-known that improved fat oxidation ability is one of the most potent contributors to increased endurance exercise capacity^12^, the direct effects of the currently known changes in DHT concentration during exercise on energy substrate utilization in the whole body, that is, energy consumption and fat and carbohydrate oxidation, remain unclear.

Therefore, we hypothesized that blocking DHT hormone production during moderate-intensity treadmill exercise would inhibit the elevation in whole-body fat oxidation during exercise. Accordingly, the purpose of this study was to determine the effects of reduced DHT activity during running exercise in a metabolic chamber on whole-body energy consumption and fat and carbohydrate oxidation by blocking DHT production in ICR male mice during one-time moderate-intensity treadmill exercise.

## METHODS

### Subjects

Twelve 9-week-old mature male ICR mice were purchased and acclimatized to the feeding environment for 1 week while providing experimental animal feed. All mice were acclimatized in standardized plastic cages with consistent humidity (50%) and temperature (23 ± 1°C). Drinking water and unrefined standardized feed were provided (5L79, Orient Bio, Inc., Seongnam, Korea). The ratio of carbohydrate, protein, and fat in the feed was 65:21:14.

### Experimental design

After acclimatization, the 10-week-old mice were randomly assigned to the control group (CON, *n* = 6) and control + DHT inhibitor group (CONIN, *n* = 6). Some mice used in our previous study^13^ were used in the present study. The CONIN group was administered a previously prepared SRD5A1A2 inhibitor (Avodart, dutasteride 2mg/kg, 1ml) diluted in lipid-soluble fat in the subcutaneous fat around the scapula at 2 days, 1 day, and 1 h prior to onetime moderate-intensity exercise^14^. The CON group were administered the same amount of vehicle (corn oil 1ml) at the same time points. Exercise was performed after fasting for 12 h overnight.

### Exercise treatment

One-time moderate-intensity aerobic exercise at 60–70% VO_2max_ and 25 m/min was performed for 30 min on a treadmill at 3° in a gas chamber that enables the measurement of energy metabolism in rodents^13^. The mice that experienced electric stimulation 2–3 times earlier in the exercise completed the 30-min exercise without additional stimulation, and all 12 mice completed the exercise.

### Gas analysis

The two groups of mice were placed in the energy metabolism calorimeter chamber to measure energy metabolism during exercise for 30 min^15^. Metabolic gas was measured using an open-circuit apparatus with reference to previous studies^2,4^. O_2_ uptake and CO_2_ production were analyzed using a mass analyzer (model RL-600, Alco System, Chiba, Japan; using a gas analyzers) and switching system (model ANI6-A-S, Alco System). O_2_ uptake and CO_2_ production were used to calculate the respiratory exchange ratio (RER, VCO_2_/VO_2_) and fat and carbohydrate oxidation^16^.

### Data processing

The collected data were analyzed using SPSS statistics version 23.0 software (SPSS, Inc., Chicago, IL, USA). For descriptive statistics for all dependent variables, the mean (M) and standard error mean (SEM) were calculated, and statistical significance (α) was set to below 5%. The effects of time and group were analyzed by two-way repeated measures analysis. Further, the area under the curve (AUC) was used to predict the amounts of energy consumed and substrate oxidation during exercise, and the two groups were compared using an unpaired t-test.

## RESULTS

**Fig. 1.**
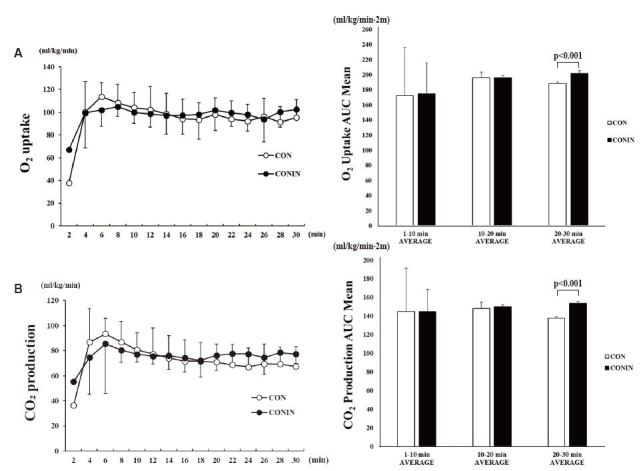
Change in O_2_ uptake (A) and CO_2_ production (B) during running exercise for 30 min. Two-way ANOVA result (A): Time, 0.667; Group, 0.597; Interaction, 0.840. Two-way ANOVA result (B): Time, 0.691; Group, 0.457; Interaction, 0.462. CON, no treatment with exercise; CONIN, DHT inhibitor treatment with exercise; AUC, area under the curve. Data are expressed as the mean ± SD.

There were no significant differences in oxygen uptake during the 30 min of exercise between the two groups (Figure 1-A). However, the AUC of oxygen uptake was significantly higher in the CONIN group (201.77 ± 3.89 mL/kg/min∙2m) than in the CON group (188.14 ± 2.32 mL/kg/min∙2m) from 20 min into exercise (*p* < 0.001). The two groups did not significantly differ in CO_2_ production during the 30 min of exercise, but the AUC was significantly higher in the CONIN group (153.90 ± 1.52 mL/kg/min∙2m) than in the CON group (137.54 ± 1.60 mL/kg/min∙2m) from 20 min into exercise (*p* < 0.001) (Figure 1-B).

**Fig. 2.**
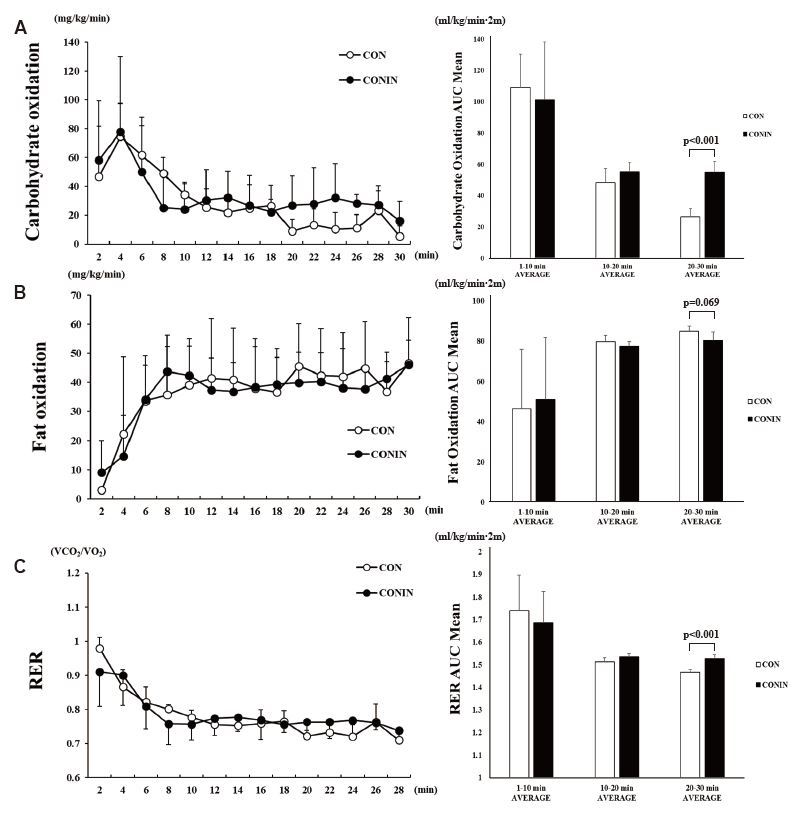
Change in carbohydrate oxidation (A), fat oxidation (B) and RER (C) during running exercise for 30 min. Two-way ANOVA result (A): Time <0.001; Group, 0.450; Interaction, 0.152. Two-way ANOVA result (B): Time <0.05; Group, 0.870; Interaction, 0.691. Two-way ANOVA result (C): Time <0.01; Group, 0.690; Interaction, 0.345. CON, no treatment with exercise; CONIN, DHT inhibitor treatment with exercise; AUC, area under the curve; RER, respiratory exchange ratio. Data are expressed as the mean ± SD.

The two groups did not significantly differ in carbohydrate oxidation during the 30 min of exercise (Figure 2-A). However, the AUC of carbohydrate oxidation was significantly higher in the CONIN group (55.02 ± 6.94 mg/kg/min∙2m) than in the CON group (26.29 ± 5.38 mg/kg/min∙2m) from 20 min into exercise (*p* < 0.001). The two groups also did not significantly differ in fat oxidation during the 30 min of exercise (Figure 2-B), but the AUC of fat oxidation was lower in the CONIN group (80.21 ± 4.35 mg/kg/min∙2m) than in the CON group (84.94 ± 2.54 mg/kg/min∙2m) from 20 min into exercise (*p* = 0.067). The two groups did not significantly differ in RER during the 30 min of exercise (Figure 3-C). However, the AUC of RER was significantly higher in the CONIN group (1.526 ± 0.018 VCO_2_/VO_2_∙2m) than in the CON group (1.466 ± 0.013 VCO_2_/VO_2_∙2m) from 20 min into exercise (*p* < 0.001).

## DISCUSSION

When treated with an inhibitor of DHT production during 30-min moderate-intensity exercise, oxygen uptake and CO_2_ production increased starting at 20 min after starting the exercise. Furthermore, the DHT production-inhibited group showed significantly higher carbohydrate oxidation and RER than the control group from 20 min after starting exercise. Our results suggest that changes in DHT concentration affect endurance exercise capacity by regulating fat oxidation during exercise.

In this study, we observed that oxygen uptake and CO_2_ production increased from 20 min after starting moderate-intensity treadmill exercise when the mice were treated with an inhibitor of DHT production. Because the two groups in our study were subjected to exercise of the same intensity and duration, the elevated energy consumption during exercise as a result of inhibition of DHT production may reflect relatively greater energy consumption and lower energy efficiency. This may be partially explained by the carbohydrate and fat oxidation results in our study. Beginning at 20 min after starting the exercise, the DHT inhibition group showed approximately 5.8% lower AUC of fat oxidation (*p* = 0.067), but approximately 109% higher carbohydrate oxidation (*p* < 0.001), revealing that the difference in carbohydrate oxidation was greater than that in fat oxidation. The specific reason for this is unknown, but we cautiously think that the DHT inhibition group consumed relatively more energy at later times during exercise than the control group because of higher energy consumption via carbohydrate oxidation, although fat oxidation did not significantly differ between groups.

In this study, the DHT inhibition group showed a higher RER than that the control group starting 20 min after beginning exercise. Based on this result, we speculated that changes in DHT during exercise affect fat oxidation. In a recent study by Bolduc et al.^10^, DHT was reported to increase acyl-coenzyme A dehydrogenase very long chain, which regulates beta-oxidation in fat tissues, while increasing carboxylesterase 3, monoglyceride lipase, and hormone-sensitive lipase, which regulate lipolysis in male mice. Further, Bolduc et al.^11^ reported that the expression of CD36, which transports fat to the mitochondria, was upregulated in mice administered DHT for 3 weeks. Therefore, this is the first study to suggest that DHT, an androgen hormone, is a regulator of fat energy utilization during moderate-intensity aerobic exercise.

One limitation of this study is the small sample size. Another limitation is that the CON group had a high RER (nearly 1) in the first 2 min of exercise, which can affect RER at later time points during exercise. Further studies should investigate energy metabolism at rest after administering DHT inhibitors and energy consumption during physical activities other than exercise. Sato et al.^9^ suggested that androgen hormone production increased with increasing intensity of aerobic exercise, and thus studies should also examine the degree of DHT hormone production and energy substrate utilization in relation to exercise intensity. In the present study, a moderate-intensity (60–70% VO_2max_) exercise—an intensity at which fat oxidation is activated—was used, and an exercise protocol involving an intensity and duration at which elevation of DHT concentration in muscles and adipose tissues was used^6,13^. Additional studies are needed to analyze the exhausting exercised time or distance to examine the effects of changes in DHT hormone during exercise on endurance exercise capacity.

We observed increased energy consumption at the later phase of the moderate-intensity treadmill running when DHT production was inhibited. Furthermore, when DHT production during exercise was inhibited, whole-body fat utilization was inhibited and carbohydrate oxidation was substantially increased at the later phase of the exercise compared to in the control group. Therefore, changes in DHT concentration in the body during exercise may be involved in whole-body fat utilization, suggesting that DHT may be an important factor affecting endurance exercise capacity.
